# Draft Genome Sequence of the Flagellated *Xanthomonas fuscans* subsp. *fuscans* Strain CFBP 4884

**DOI:** 10.1128/genomeA.00966-14

**Published:** 2014-09-25

**Authors:** A. Indiana, M. Briand, M. Arlat, L. Gagnevin, R. Koebnik, L. D. Noël, P. Portier, A. Darrasse, M. A. Jacques

**Affiliations:** a INRA, Institut de Recherche en Horticulture et Semences (IRHS), UMR 1345 SFR 4207 QUASAV, Beaucouzé, France; b Université d’Angers, Institut de Recherche en Horticulture et Semences (IRHS), UMR 1345 SFR 4207 QUASAV, Beaucouzé, France; c Agrocampus Ouest, Institut de Recherche en Horticulture et Semences (IRHS), UMR 1345 SFR 4207 QUASAV, Beaucouzé, France; d INRA, Laboratoire des Interactions Plantes Micro-Organismes (LIPM), UMR 441, Castanet-Tolosan, France; e CNRS, LIPM, UMR 2594, Castanet-Tolosan, France; f Université de Toulouse, Université Paul Sabatier, Toulouse, France; g UMR Peuplements Végétaux et Bioagresseurs en Milieu Tropical (PVBMT), CIRAD, Saint-Pierre, La Réunion, France; h UMR 186 IRD-Cirad-Université Montpellier 2 “Résistance des Plantes aux Bioaggresseurs,” Montpellier, France

## Abstract

We report the draft genome sequence of the flagellated strain CFBP 4884 of *Xanthomonas fuscans* subsp. *fuscans*, which was isolated in an outbreak of common bacterial blight of beans along with non-flagellated strains. Comparative genomics will allow one to decipher the genomic diversity of strains cohabiting in epidemics.

## GENOME ANNOUNCEMENT

*Xanthomonas fuscans* subsp. *fuscans* is one of the causal agents of the common bacterial blight of beans (*Phaseolus vulgaris*) (1). This disease is distributed worldwide where beans are cultivated, except in arid tropical regions (2). Bacterial blight of beans is the most devastating bacterial disease of beans (3) which can cause a 40% yield loss (2). Seed contamination impacts both bean production and the seed industry worldwide through direct and indirect costs.

*Xanthomonas* spp. are γ-proteobacteria motile by a single polar flagellum (4). Motility is an important feature involved in plant colonization and is often considered to be a pathogenicity factor. However, the only genome sequence available of *X. fuscans* subsp. *fuscans* was obtained from a non-flagellated strain (5). In this strain, a mobile element, ISXfu2, is associated with a deletion of 33 kb in the flagellar gene cluster, thus affecting the biosynthesis of the flagellum and motility. However, motility was identified in 95% of the tested *X. fuscans* subsp. *fuscans* strains. Some of the motile strains of *X. fuscans* subsp. *fuscans* were isolated from the same epidemic and the same field as the non-flagellated strain CFBP 4834 (5). Hence, to better understand if the absence of flagellation is linked to a recent genetic event in a genetically homogeneous population or, in contrast, to the cohabitation of strains having diverged in the past, we sequenced the genome of the flagellated strain CFBP 4884 of *X. fuscans* subsp. *fuscans*, which was isolated along with strain CFBP 4834 from a bean leaf in 1998 in a heavily infected bean field in Beaucouzé, France (6). This strain is highly aggressive on beans.

The genome was sequenced using the Illumina Hi-Seq2500 platform (Genoscreen, France) The shotgun sequencing yielded 5,954,190 read pairs (100-bp paired-end reads with insert size of 1200 bp). A combination of Velvet (7), SOAPdenovo, and SOAPGapCloser (8) yielded 203 contigs larger than 200 bp (*N*_50_ = 79,889 bp) with the largest contig of 319,482 bp for a total assembly size of 5,003,118 bp. Genomic contigs were annotated using the EugeneP annotation pipeline to identify RNAs and protein-coding genes (9).

The genome of the strain CFBP 4884 has a gene content similar to other xanthomonads, and is most similar to the genome of the strain 4834-R (5). The exceptions are a set of 31 genes of the flagellar gene cluster, which are absent in the strain 4834-R and present in the CFBP 4884 genome. Fifteen other genes, coding hypothetical proteins and adhesins, are divergent between these two genome sequences. This resource will be valuable to investigate the mechanisms leading to the emergence of non-flagellated strains and to dissect the genomic diversity of strains of a quarantine pathogen.

### Nucleotide sequence accession numbers.

This whole-genome shotgun project has been deposited at DDBJ/EMBL/GenBank under the accession no. JPHG00000000. The version described in this paper is the first version, JPHG01000000.
